# A Freeze Frame View of Vesicular Stomatitis Virus Transcription Defines a Minimal Length of RNA for 5′ Processing

**DOI:** 10.1371/journal.ppat.1002073

**Published:** 2011-06-02

**Authors:** Gergely Tekes, Amal A. Rahmeh, Sean P. J. Whelan

**Affiliations:** Department of Microbiology and Molecular Genetics, Harvard Medical School, Boston, Massachusetts, United States of America; Centro Nacional de Biotecnologia (CSIC) and CIBER de Enfermedades Respiratorias, Spain

## Abstract

The RNA synthesis machinery of vesicular stomatitis virus (VSV) comprises the genomic RNA encapsidated by the viral nucleocapsid protein (N) and associated with the RNA dependent RNA polymerase, the viral components of which are a large protein (L) and an accessory phosphoprotein (P). The 241 kDa L protein contains all the enzymatic activities necessary for synthesis of the viral mRNAs, including capping, cap methylation and polyadenylation. Those RNA processing reactions are intimately coordinated with nucleotide polymerization such that failure to cap results in termination of transcription and failure to methylate can result in hyper polyadenylation. The mRNA processing reactions thus serve as a critical check point in viral RNA synthesis which may control the synthesis of incorrectly modified RNAs. Here, we report the length at which viral transcripts first gain access to the capping machinery during synthesis. By reconstitution of transcription in vitro with highly purified recombinant polymerase and engineered templates in which we omitted sites for incorporation of UTP, we found that transcripts that were 30-nucleotides in length were uncapped, whereas those that were 31-nucleotides in length contained a cap structure. The minimal RNA length required for mRNA cap addition was also sufficient for methylation since the 31-nucleotide long transcripts were methylated at both ribose-2′-O and guanine-N-7 positions. This work provides insights into the spatial relationship between the active sites for the RNA dependent RNA polymerase and polyribonucleotidyltransferase responsible for capping of the viral RNA. We combine the present findings with our recently described electron microscopic structure of the VSV polymerase and propose a model of how the spatial arrangement of the capping activities of L may influence nucleotide polymerization.

## Author Summary

Using a prototype of the nonsegmented negative strand RNA viruses, vesicular stomatitis virus, we probed the spatial relationship between the RNA dependent RNA polymerase and 5′ mRNA capping and methylation activities of the large polymerase protein. Because the 5′ mRNA processing reactions dramatically impact the nucleotide polymerization activity of the protein, they may function as a quality control step in viral transcription. We developed a means to stall transcription at precisely defined locations following initiation and analyzed the cap status of the stalled transcripts. We show that 30-nt transcripts are uncapped whereas those that are 31-nt long gain are capped and methylated at both guanine-N-7 and ribose-2′-O positions. Combined with our recent work that determined the molecular architecture of the VSV polymerase, this work reveals the spatial relationship within a functional polymerase complex of the polymerase domain and the 5′ mRNA processing domains of the L protein.

## Introduction

The RNA synthesis machinery of the non-segmented negative-strand (NNS) RNA viruses contains at its core a large polymerase protein (L) that possesses all the enzymatic activities for genome transcription and replication. During transcription, L catalyzes nucleotide polymerization [1]–[3] as well as each step of mRNA cap addition [4]–[9] and polyadenylation [10]. Those activities are intimately coordinated such that failure to cap the mRNA results in the premature termination of RNA synthesis [7], [11]–[13], and failure to methylate the mRNA can result in the hyper polyadenylation of the mRNA [13]–[15]. In this study, we sought to examine how the different L activities are coordinated to ensure the correct synthesis of a capped and methylated mRNA by precise determination of the point at which those 5′ mRNA processing reactions occur during transcription.

Our understanding of the activities of L protein has been largely shaped by studies of a prototype of the NNS RNA viruses, vesicular stomatitis virus (VSV). The NNS RNA virus L proteins are homologous and share six regions of sequence conservation (CRI-VI) [16] that were thought to contain the conserved functions. The RNA dependent RNA polymerase (RdRP) was readily identified by the presence of a set of motifs in CRIII [1]. Consistent with this assignment, substitution of an aspartic acid residue predicted to coordinate a catalytically essential magnesium ion ablates nucleotide polymerization *in vitro*. Although L protein was known to possess the enzymatic activities for mRNA cap addition, their identity proved difficult to pin down unambiguously. This is because the enzymatic activities themselves are unusual, in that the cap is added by the action of a polyribonucleotidyltransferase (PRNTase) that transfers pRNA onto a GDP acceptor through a covalent L-pRNA intermediate [7], [9], [17] This contrasts with all other known capping reactions which involve an RNA guanylyltransferase that transfers GMP onto a diphosphate RNA acceptor [18]. Substitutions to residues in CRV of VSV L that are conserved throughout all NNS RNA viruses led to the ablation of capping activity *in vitro* and defined a motif GxxT[N]HR that was essential for capping, implicating CRV as the PRNTase [7]. Subsequently, the conserved histidine was shown to be essential to form the covalent L-pRNA intermediate, further substantiating this assignment [17]. Polymerases that were defective in mRNA cap addition terminated transcription prematurely, further underscoring the link between correct 5′ mRNA processing and nucleotide polymerization [7].

Following cap formation, the cap itself is methylated at guanine-N-7 and ribose-2′-O positions [5], [19]–[25]. Those reactions also differ for VSV since they are catalyzed by a single methyltransferase domain [8]. Moreover, in contrast to the order of cap methylation events in other viruses and organisms, the ribose 2′-O methylation precedes and facilitates the subsequent guanine-N-7 methylation [26]. Sequence alignments identified a methylase like domain in CRVI of L [27], [28], and substitutions within this region block both 2′-O and G-N-7 methylation [6], [8]. Like capping, the act of methylation can also influence the properties of the RdRP, since in some circumstances a failure to methylate the mRNA cap is accompanied by the production of a large polyadenylate tail by excessive stuttering of the polymerase on a U7 tract [13]–[15]. This intimate co-ordination of the 5′ mRNA processing reactions with nucleotide polymerization may help ensure production of correctly modified transcripts, which in turn could minimize triggering of cellular pathways that recognize either uncapped or unmethylated RNA [29].

Recently, we obtained a first view of the molecular architecture of the VSV L polymerase protein [30]. Single particle electron microscopy revealed that the capping machinery of L resides within 3 globular domains that are appended to a core ring-like RdRP domain. Moreover the architecture of L rearranges significantly following complex formation with the essential viral polymerase cofactor P, and this rearrangement likely positions the domains in the correct orientation to ensure the modification of the nascent mRNA chain [30]. In this study, we sought to determine at what stage a VSV mRNA acquires a cap structure. Since the PRNTase and the RdRP are localized within different regions of the L protein, a minimal length of RNA may serve as an important check point regulating the distinct activities. For decades RNA synthesis reactions have been carried out *in vitro* with VSV using either virus in which the membrane is disrupted with detergent [31], or purified polymerase (L and P) and the N-RNA template [32], [33]. From those reactions, the shortest transcripts that were identified as being capped were 37-nucleotides [34]. It was not clear, however, whether those transcripts were capped during synthesis or had at some level gained access to the PRNTase following release from the polymerase. Recent experiments have shown that short 5-nt transcripts corresponding to the beginning of a VSV mRNA can be capped *in trans* by L, but such experiments cannot address at what stage during transcription the RNA chain is modified. In the present study, we established a system to provide a “freeze-frame” view of VSV transcription using templates that lacked sites for UTP incorporation. By stalling transcription reactions at precisely defined chain lengths we identify a minimal length at which the transcript becomes capped. This work reveals the spatial arrangements of the capping activities of the VSV L protein in relation to the RNA dependent RNA polymerase domain during active transcription.

## Results

### Generation of recombinant viruses to permit stalling of transcription at specific locations

**Figure 1:**
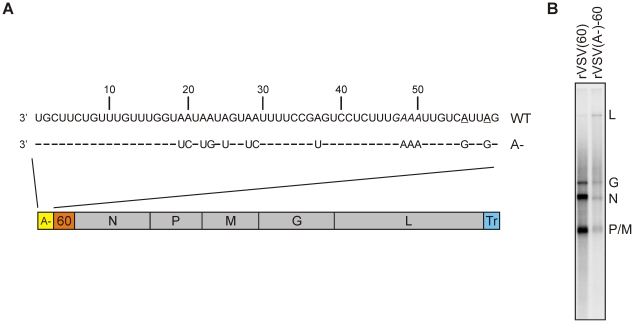
Effect of an (A-) leader region on transcription. (A) Schematic of the VSV genome containing an (A-) leader region and a 60-nt long gene inserted between the leader region and the N gene. The complete sequence of the leader region is shown. The positions at which G residues were engineered to replace the original A residues in the gene-start sequence are underlined (B) *In vitro* transcription reactions using 10 µg of each of the purified viruses were performed in the presence of [α-^32^P]-GTP. Purified RNA was analyzed on acid-agarose gel and detected using a phosphorimager. The identity of the mRNAs is shown on the right.

The promoter for VSV mRNA synthesis includes the 3′ leader region and the first 10-nt of the conserved gene-start element [35]–[38]. The sequences of those elements specify the incorporation of each of the four NTP's into the nascent RNA chain (Figure 1A), creating a challenge in selecting a nucleotide that can control polymerase stalling. We elected to engineer the template such that it lacked sites for UTP incorporation. We chose UTP because of the known requirement for a high ATP concentration for initiation of RNA synthesis, and because previous work had revealed the essential nature of sites for CTP and GTP incorporation in the cis-acting signals in the mRNA start sequence [11], [39]. To do this, we engineered the leader region and the gene-start element of a non-essential 60-nt gene that was inserted at the leader-N gene junction of an infectious cDNA clone of VSV (Figure 1A). Specifically, we modified the leader sequence such that it lacked adenosine nucleotides except for those at position 48–50 which are typically not transcribed by the polymerase. We generated recombinant viruses in which those mutations were engineered within the leader region, purified the virus and confirmed that the mutations were present in the viral genome (data not shown).

To examine the effect of the leader mutations on viral transcription, we performed transcription reactions *in vitro*. Briefly, 10 µg of purified virus was incubated with detergent to disrupt the viral membrane and NaCl to liberate the M protein from the RNP core and permit transcription. The detergent-disrupted particles were incubated in transcription buffer containing ATP, CTP, UTP and [^32^P]-GTP and the products of transcription purified and analyzed by electrophoresis on acid-agarose gels (Figure 1B). Although overall levels of transcription were reduced (compare rVSV (60) and rVSV(A-)60), indicating that the U- leader promoter was less efficient than the wild type sequence, each of the viral mRNAs were synthesized. This result demonstrates that the sequence of the viral leader region could be engineered to eliminate sites of UTP incorporation which forms the basis of the templates to stall transcription within the downstream 60-nt transcriptional unit.

**Figure 2:**
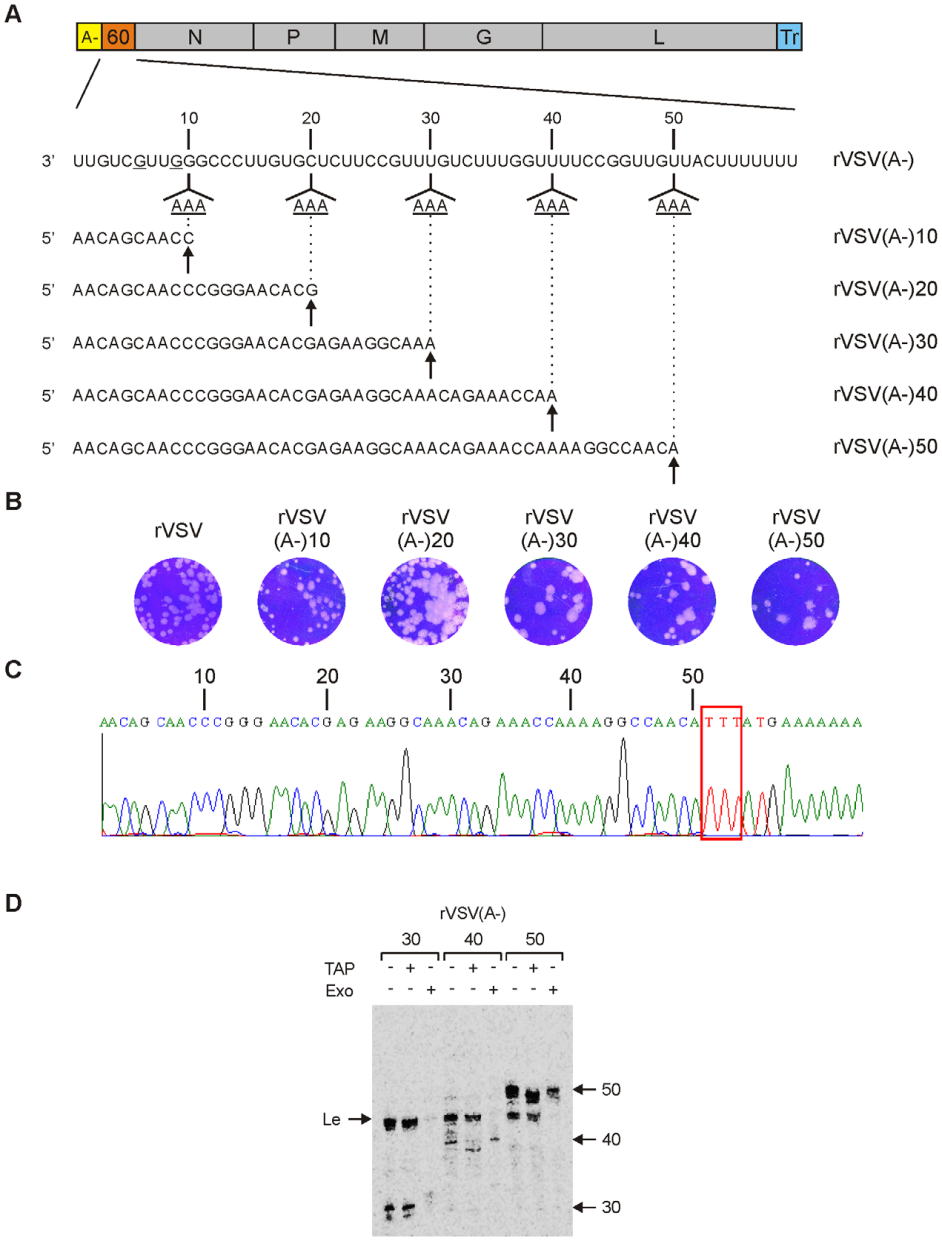
Recombinant VSV lacking sites for UTP incorporation permit stalling of transcription. (A) Schematic of the VSV genome containing an (A-) leader region and a 60-nt long gene inserted between the leader region and the N gene. The complete sequence of the 60-nt gene is shown, and positions at which G residues were engineered to replace the original A residues in the gene-start sequence are underlined. The sites (11, 21, 31, 41 and 51) at which 3 sequential adenylates were inserted within the 60-nt gene are indicated together with the anticipated sequence of the corresponding stalled transcript. (B) The plaque morphology is shown at 48 h postinoculation for rVSV(A-)-10, -20, -30, -40 and -50 and at 24 hpi for rVSV. (C) A sequence trace of the genomic RNA derived from the 60-nt gene of rVSV(A-)-50 is shown to illustrate the three introduced sites of UTP incorporation. (D) The indicated recombinant VSV were used in *in vitro* transcription (IVT) reaction lacking UTP, but containing [α-^32^P]-GTP. The products of transcription were analyzed on a 6% polyacrylamide gel and visualized using a phosphorimager. Where indicated, the products of transcription were first digested with tobacco acid pyrophosphatase (TAP) which hydrolyzes the cap-structure, or with an exonuclease (Exo) that digests uncapped RNA. The identity of the purified viruses used for the IVT reactions are shown at the top of the gel. The uncapped leader RNA and the 30, 40 and 50-nt long transcripts are indicated. A representative gel of two independent experiments is shown.

The 60-nt gene sequence was next modified such that the first place the polymerase would encounter sites for UTP incorporation were at positions +11–13, 21–23, 31–33, 41–43, or 51–53 with respect to the first gene-start (Figure 2A). Infectious recombinant VSV was recovered from each of those clones and while all the mutants grew less well than wild type VSV, there we no obvious differences in the plaque morphologies of the mutants (Figure 2B). Sequencing of the genomic RNA confirmed that the first sites for UTP incorporation were at the desired location (Figure 2C and data not shown). To determine whether transcription could be stalled at the inserted adenylates, we used purified recombinant viruses and performed RNA synthesis in the absence of UTP. Briefly, 10 µg of purified virus was disrupted with detergent, incubated in transcription buffer containing ATP, CTP and [^32^P]-GTP and the products of transcription purified and analyzed by electrophoresis on polyacrylamide gels. The VSV recombinants that were designed to stall transcription at positions +30, 40 and 50, generated RNA of the anticipated length, thus demonstrating that transcription can be specifically stalled by omission of UTP from the reactions (Figure 2D). We did not observe significant quantities of a read-through transcript where polymerase incorporated another nucleotide in place of UTP to generate a full-length 60-nt transcript (Figure 2D). This indicates that the polymerase error rate is insufficient to bypass three sites of UTP incorporation through mis-incorporation of an alternate nucleotide.

Treatment of the RNA products with the cap cleaving enzyme tobacco acid pyrophosphatase (TAP) revealed that the stalled 30-nt transcript was insensitive to cleavage as evidenced by its unaltered mobility, whereas the 40-nt and 50-nt transcripts shifted in mobility by a single nucleotide following cleavage (Figure 2D). This indicates that the 30-nt transcripts were uncapped, whereas the 40 and 50-nt long transcripts were capped. Using a 5′-3′ exonuclease (+Exo) that cleaves uncapped RNA, we further confirmed that the 40-nt and 50-nt transcripts were capped, since those RNAs were resistant to digestion, whereas the 30-nt transcript was sensitive (Figure 2D). As expected, each of the recombinant viruses also produced a leader RNA as evidenced by the collection of transcripts around 47-nt (Figure 2D). Leader RNA synthesized *in vitro* is known to contain at least 4 distinct 3′ termini which likely accounts for the multiple bands observed for the altered leader sequence [40]. Those leader transcripts lack an mRNA cap structure as shown by their insensitivity to cleavage by TAP, and their hydrolysis by the 5′-to 3′ exonuclease. Collectively, this analysis indicates that a VSV mRNA gains access to the mRNA capping machinery at an RNA chain length of >30-nt, but <40-nt. This experiment, however, could not determine whether the RNA was capped within a range of 30–40 nt or whether capping required a specific chain length.

### VSV mRNA gains access to the mRNA capping machinery at a precisely defined nucleotide length

**Figure 3:**
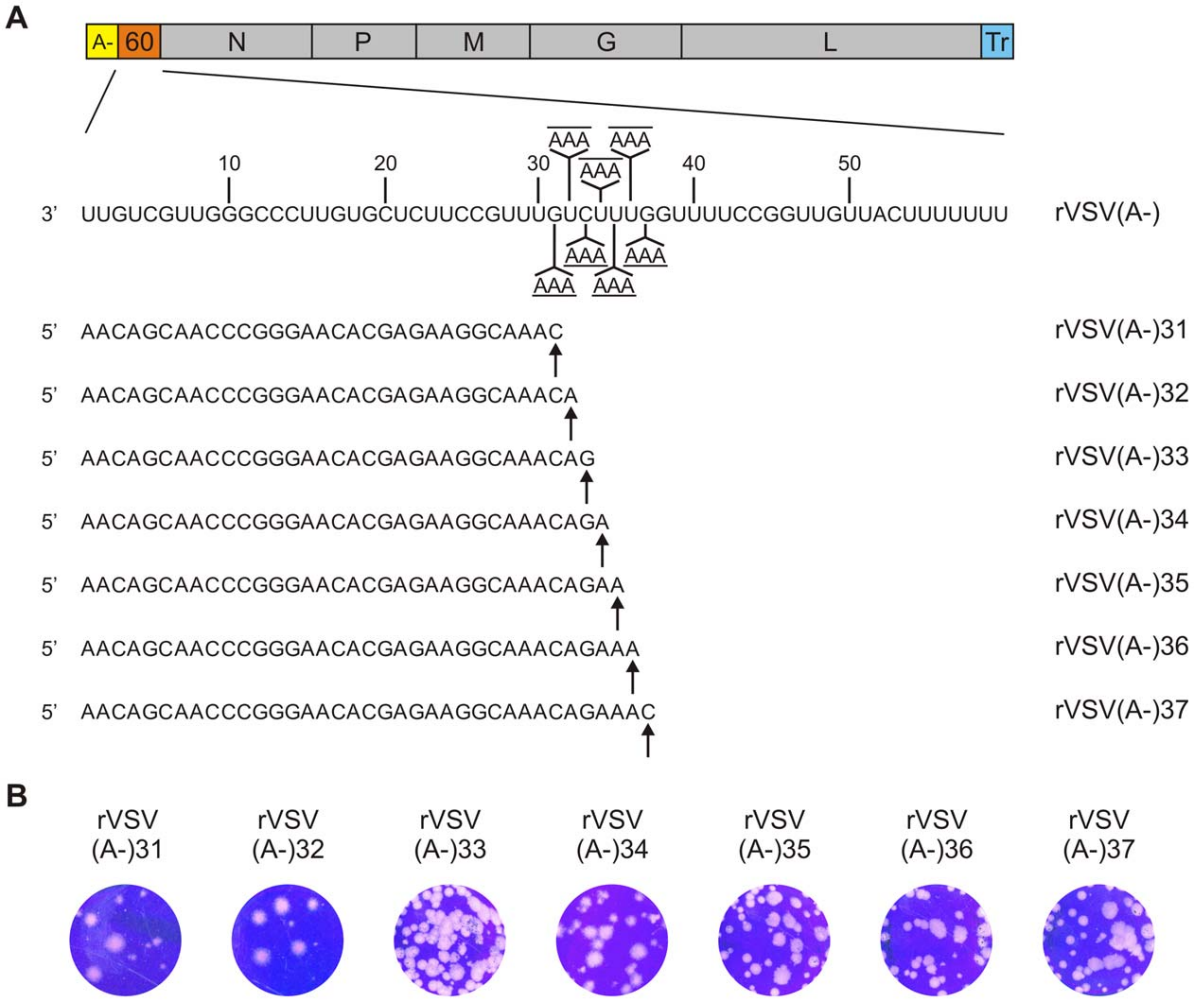
Generation and characterization of recombinant VSV. (A) Schematic representation of the genome organization of rVSV(A-)60. The complete sequence of the 60-nt non-essential gene is shown. The positions (32, 33, 34, 35, 36, 37 and 38) of the introduced UTP incorporation sites in the 60-nt gene are indicated. The estimated sequences of the different stalled transcripts are shown. (B) Plaque morphology of rVSV(A-)-31. -32, -33, -34, -35, -36 and -37 is shown at 48 hpi.

**Figure 4:**
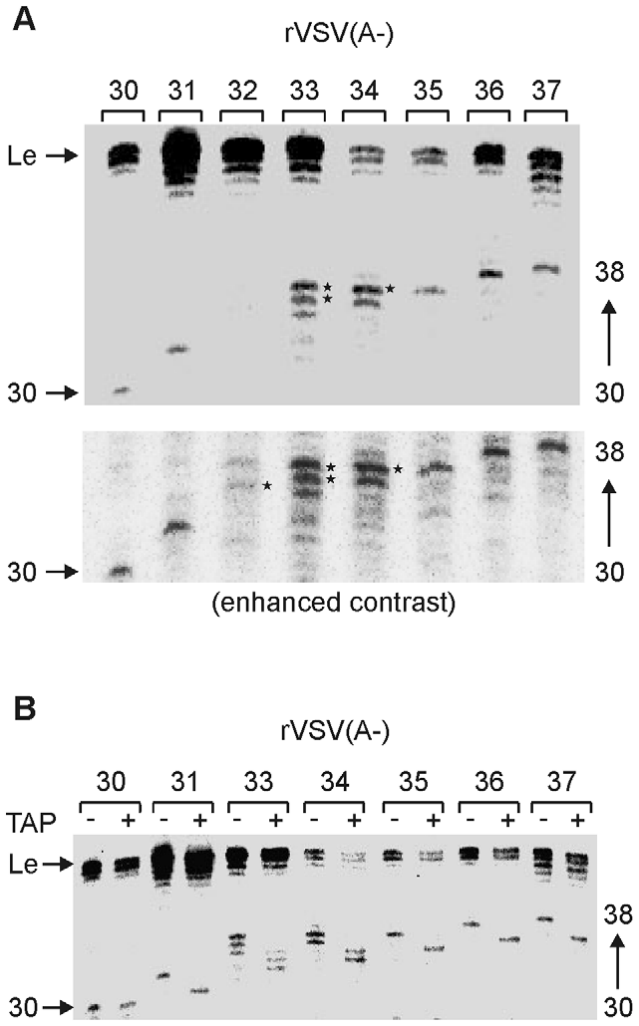
Determination to a single nucleotide the length at which VSV mRNA gets capped. (A) An autoradiograph of a 6% polyacrylamide gel is shown indicating the products of the IVT reactions with recombinant VSV containing a 60-nt long non-essential gene that lacks sites for UTP incorporation except at positions 31, 32, 33, 34, 35, 36, 37 or 38 respectively. The reaction was performed in the absence of UTP, but containing [α-^32^P]-GTP. The identity of the purified viruses used for the IVT reactions are shown at the top panel. The uncapped leader RNA and the different transcripts between 30 and 38 nt are indicated. Asterisks indicate the 1–2 nt longer than anticipated transcripts of the rVSV(A-)-33 and -34 viruses. (B) Recombinant VSV(A-)-30, -31, -33, -34, -35, -36 and -37 were used in *in vitro* transcription (IVT) reactions lacking UTP, but containing [α-^32^P]-GTP. The products were analyzed on 6% polyacrylamide gel and visualized using a phosphorimager. The products of transcription were either untreated (-) or digested with TAP (+) which cleaves the cap-structure. The identity if the purified viruses used for the IVT reactions are shown at the top panel. The uncapped leader RNA and the different transcripts between 30 and 38 nt are indicated. A representative gel from two independent experiments is shown.

To more precisely define the point at which the RNA chain gains access to the capping machinery, we constructed a set of recombinant viruses designed to stall transcription at intermediate points between 30 and 40-nt (Figure 3A). Specifically, we recovered viruses designed to stall transcription at 31–37 nts (Figure 3B) and examined the products made by those viruses following transcription reactions performed as above. As expected, each of those viruses generated the characteristic profile of leader RNAs around 47-nt, as well as specifically stalled transcripts (Figure 4A). The mobility of the majority of the transcripts matched the anticipated position of stalling as shown by those RNAs that were 30, 31, 35, 36 and 37-nt long (Figure 4A). The mobility difference between the transcripts produced by the viruses designed to stall transcription at 30 vs. 31-nt appears to be 2-nt, indicating that the 31-nt transcript was fully capped. By contrast, stalling appeared relatively inefficient and somewhat heterogeneous for the viruses designed to stall transcription at positions +33 and +34 extending from the anticipated length (indicated by the *) to 1–2 nt larger. Stalling of transcription at +32 appeared inefficient with only low levels of the stalled transcript visible on the gel (Figure 4A). An enhanced contrast view of the gel is provided to illustrate the low levels of stalled transcripts obtained with the virus designed to stall transcription at +32 (Figure 4A, lower). Although we do not know the reason for this altered stalling efficiency it correlates precisely with the point immediately following mRNA cap addition. The ratio of leader to stalled transcript appears to vary for several of the recombinant viruses. We do not know the basis for this variation, but with the exception of the virus designed to stall transcription at +32, each of the viruses generated sufficient stalled transcripts for us to determine the cap status.

To definitively determine whether those transcripts contained an mRNA cap structure, we compared the mobility of the stalled transcripts before and after TAP digestion. As expected, the leader RNA, and the +30 RNA were insensitive to TAP cleavage indicating that they lack an mRNA cap structure (Figure 4B). By contrast the transcripts stalled at +31, +35, +36 and +37 showed a clear single nucleotide shift in their mobility following TAP cleavage (Figure 4B). Moreover, the collection of transcripts synthesized following stalling at positions +33 and +34 shifted in mobility indicating that they were capped (Figure 4B). Collectively, these data indicate that a VSV mRNA gains access to the capping machinery at a chain length of 31-nt.

### Reconstitution of transcription using a cap-defective polymerase confirms that the nascent RNA chain gains access to the capping machinery at +31-nt

**Figure 5:**
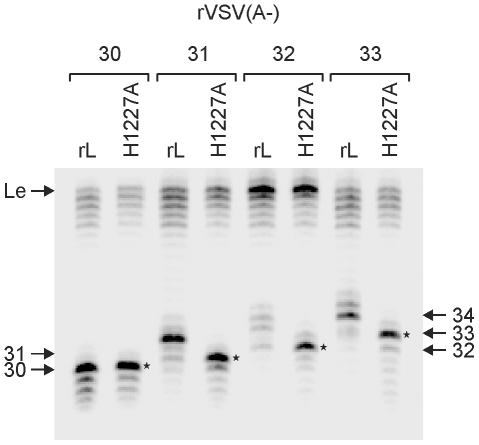
Capping occurs at a nascent RNA chain length of 31-nt. *In vitro* transcription reactions were reconstituted with 5 µg of N-RNA template, 2 µg of purified P, and 4 µg of the indicated L protein in the absence of UTP, but in the presence of [α-^32^P]-GTP. Purified RNA was analyzed on a 6% polyacrylamide gel and detected using a phosphorimager. The identity of the template and for the reactions used polymerase molecules are shown at the top the panel. The uncapped leader RNA and the different transcripts between 30 and 34 nt are indicated. Asterisks indicate the uncapped transcripts made by H1227A-L with the different templates. A representative gel from two independent experiments is shown.

The above experiments show that the transcript must be 31-nt long to be capped. To provide further support for this, we took advantage of our previously characterized cap defective polymerase (L-H1227A) [7]. As expected, the transcripts synthesized following stalling of transcription at +30 were uncapped (Figure 5), and identical products were generated by L-H1227A. Using wild type L, stalling of transcription at position +31 and above demonstrated that the transcripts were capped. Consistent with this, the cap defective polymerases generated stalled transcripts that were 1-nt shorter. This experiment confirms the identity of the uncapped stalled transcripts and further supports that a VSV mRNA gains access to the mRNA capping machinery at an RNA chain length of 31-nt. We also noted that the cap defective polymerase appears to stall more readily on the rVSV(A-)32 template. This raises the possibility that the inability to efficiently stall RNA synthesis reflects a transition of the polymerase related to cap addition.

### Further elongation is not required for cap methylation

**Figure 6:**
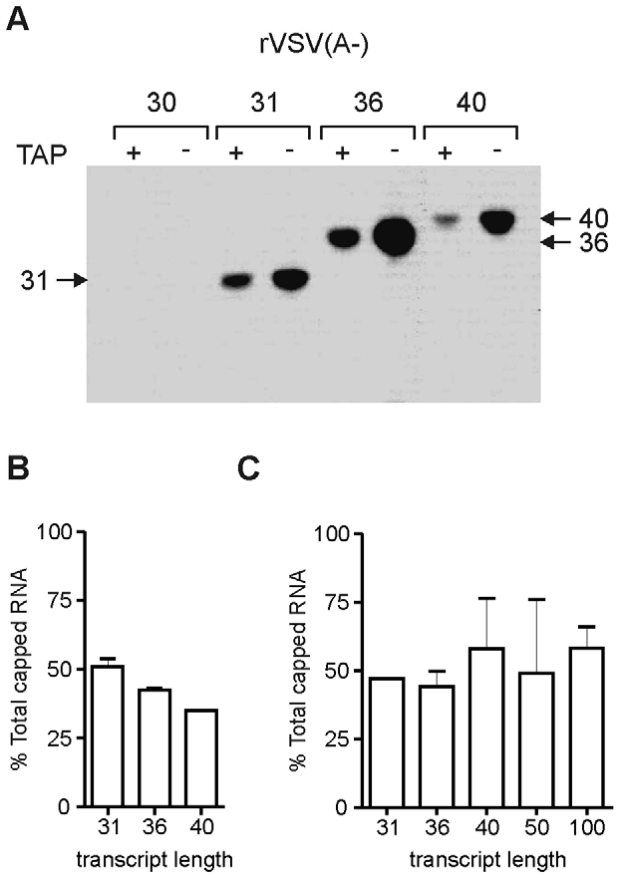
Methylation status of the stalled transcripts. (A) *In vitro* transcription reactions were reconstituted with 5 µg of N-RNA template, 2 µg of purified P, and 4 µg of the recombinant L protein in the absence of UTP, but in the presence of ^3^H-labeled S-adenosyl-L-methionine. Purified RNA was analyzed on a 20% polyacrylamide gel followed by autoradiography. The identity of the template used for the IVT reactions are shown at the top of the panel. Where indicated, the products of transcription were digested with tobacco acid pyrophosphatase (TAP) which cleaves the cap-structure. The transcripts between 31 and 40-nt are indicated. (B) The autoradiograms from two independent experiments equivalent to that shown in panel A, were analyzed by densitometric scanning and the band intensities measured. A graph showing the % of the total methylated RNA that remains following TAP cleavage is shown. (C) Total RNA from stalled *in vitro* transcription reactions performed in the presence of ^3^H-SAM was quantified by scintillation counting before and after TAP cleavage. A graph showing the % of total radioactivity measured by scintillation counting following TAP cleavage of the purified RNA is shown.

Electron microscopic structural analyses of VSV L indicate that the capping and cap methylation activities reside within distinct globular domains that are connected to a ring-like domain containing the RdRP. The capping enzyme resides in a globular domain that is proximal to the RdRP, whereas the MTase located to a more distal globular domain. The position of the MTase domain showed a degree of variability suggesting a high degree of flexibility within L. Significant structural rearrangements occur in L, following complex formation with the essential cofactor P rendering a straightforward identification of the capping and MTase domains within the active polymerase complex difficult. We therefore sought to determine whether there was an additional length requirement for mRNA cap methylation. To do this, we performed transcription reactions on the engineered templates in the absence of UTP and the presence of [^3^H]-SAM. The resulting transcripts were purified, analyzed by electrophoresis on a 20% polyacrylamide gel and detected by fluorography (Figure 6A). The 30-nt uncapped transcripts were not labeled by [^3^H]-SAM, demonstrating that lacked methyl groups at both ribose 2′-O and G-N-7 positions of the cap structure. By contrast the 31-nt and larger transcripts were labeled by [^3^H]-SAM. To evaluate whether a methyl group was present at both G-N-7 and 2′- O positions, the transcripts were exposed to TAP prior to gel electrophoresis. Following TAP cleavage, approximately 50% of the label remained associated with the transcript (Figure 6B), consistent with the removal of the 7^m^Gp structure. Because this reduction in intensity of labeling was apparent starting at the +31 transcript, the results suggest that there is no distinct length requirement for 2′-O and guanine-N-7 methylation. To quantify this effect further, we measured the amount of label associated with purified RNA before and after TAP cleavage by scintillation counting. To eliminate the released 7^m^Gp we purified RNA that was longer than 20-nt prior to scintillation counting. This analysis demonstrates that 50% of the [^3^H]-SAM signal is lost upon TAP cleavage, and indicates that the RNA cap structures are guanine-N-7 and ribose 2′-O methylated. Collectively, these data indicate that the RNA chain gains access to the MTase domain at the same length as the PRNTase domain.

## Discussion

In this study, we established a freeze-frame approach to stall VSV transcription using engineered templates that lack sites for UTP incorporation. Using this approach we defined a nascent RNA chain length requirement of 31-nucleotide for the access of the 5′ end to the mRNA capping machinery. Since the RNA chain length for mRNA cap addition is +31-nt and the distance between adjacent phosphates is approximately 3.4 Å, the physical distance that the nascent RNA chain must transit is approximately 100 Å. The requirement for a minimal length for mRNA capping underscores an architectural arrangement of VSV L where a defined distance separates the capping domain from the RdRP. The finding that the capped mRNA does not require further elongation to be methylated is consistent with a more flexible positioning of the methyltransferase domain. We discuss the present observations in light of our earlier work on the molecular architecture of the VSV polymerase, as well as previous functional studies of the impact of capping and cap methylation on mRNA synthesis. Since there are commonalities among the mechanisms of RNA synthesis, this work also likely has implications for the large polymerase proteins of other nonsegmented negative-strand RNA viruses.

### Length requirement for mRNA cap addition

The evidence presented here shows that a VSV mRNA must be 31-nt long to gain access to the mRNA capping machinery. This conclusion comes from stalling transcription at precisely that point and evaluating whether the RNA contains an mRNA cap structure. In these experiments, however, the nascent RNA chain remains stalled at 31-nt and the relative time at which the cap is added is not certain. Thus while our experiments demonstrate that the RNA chain can gain access to the PRNTase once it reaches 31-nt, they cannot determine over what range during transcription cap addition actually occurs. This will be impacted, for example, by the rate at which the PRNTase catalyzes transfer of the RNA onto GDP, and indeed the formation of the GDP acceptor. Consequently, it seems likely that cap addition occurs over a range of nucleotide positions and that the earliest possible point of capping is position +31. We and others have reported the existence of abortive uncapped transcripts that range in size up to several 100 nucleotides [7], [11], [12]. We anticipate, however, that since failure to cap leads to premature termination of mRNA synthesis, it seems likely that capping typically occurs within a short window of +31. Consistent with this latter idea it is possible to generate a short capped and polyadenylated transcript from an artificial transcription unit of 60-nt inserted between the leader and N genes of VSV [41]. The presence of uncapped transcripts that range in size up to several 100 nucleotides would then simply reflect the variable termination of transcripts that failed to gain access to the capping apparatus within the optimal window for cap addition. Our study defines the lower limit of that optimal window as 31-nt during transcription. We do not precisely know the upper limit of the window for capping nascent RNA, but since we do not observe large quantities of an uncapped 60-nt mRNA transcript synthesized in vitro [41] it seems likely that the window is quite narrow.

The finding that the transcript gains access to the methyltransferase active site at the same length, +31-nt, demonstrates that there is no further elongation requirement for cap methylation. An earlier study that examined the kinetics of mRNA cap methylation catalyzed by the VSV New Jersey polymerase provided evidence that cap methylation occurs over a nascent RNA chain length window that spanned several 100 nucleotides [42]. It seems likely, therefore, that during transcription the nascent RNA chain continues to grow while the RNA is being capped and methylated and that the precise point at which the chain gets methylated is not critical. Such a scenario therefore implies that the influence of methylation on mRNA polyadenylation, where failure to methylate can result in the production of large polyadenylate tails, is unlikely to be a coupling of the two reactions directly. Rather, the act of methylation itself in some way influences the extent of polymerase stuttering on the U7 tract present at the viral gene-end sequence and not by influencing the cessation of stuttering during the reiterative transcription process. The effects of several mutations in L that ablate mRNA cap methylation were previously examined for their ability to promote hyper polyadenylation. The majority of the methylation defective mutants synthesized mRNA with normal levels of polyadenylate [15]. The synthesis of large polyadenylate may instead be related to the affinity of the various L mutants for the inhibitor SAH, such that the hyperpolyadenylation phenotype may simply reflect increases in the Km for SAM binding rendering the polymerase mutants hypersensitive to levels of SAH.

Although the present study defines 31-nt as a minimal length of mRNA cap addition, it remains possible that there are additional gene-position or sequence dependent effects that may alter the precise position of cap addition for specific genes. For example, the sequence of the transcript may influence the length at which the RNA gets capped simply by influencing the elongation properties of the polymerase, or possibly by favoring specific nascent RNA structures that alter access of the nascent transcript to the capping site. If such gene specific effects occur, they may serve as a means to regulate the amount of full-length transcripts made from specific genes, since failure to cap would lead to premature termination, down-regulating specific gene expression. We have not yet succeeded in extending the freeze-frame methodology to study sequential transcription from the viral genome, consequently, further experiments will be required to investigate this possibility.

### Further insights into the domain organization of the VSV polymerase

Previous experiments have shown that short exogenous synthetic RNA oligonucleotides (5–10 nts) are of sufficient length to be capped when added to VSV L *in trans*. In the system used in the current study, where capping is evaluated co-transcriptionally, mRNAs shorter than 31 nts fail to be capped by the polymerase. These results indicate that while an exogenously added RNA is able to freely diffuse into the capping active site, the access of an endogenous nascent RNA to that site is regulated. Such a regulation is likely achieved through anchorage of the mRNA 3′ end by the RdRP domain preventing the 5′ end from accessing a physically distant capping active site.

**Figure 7:**
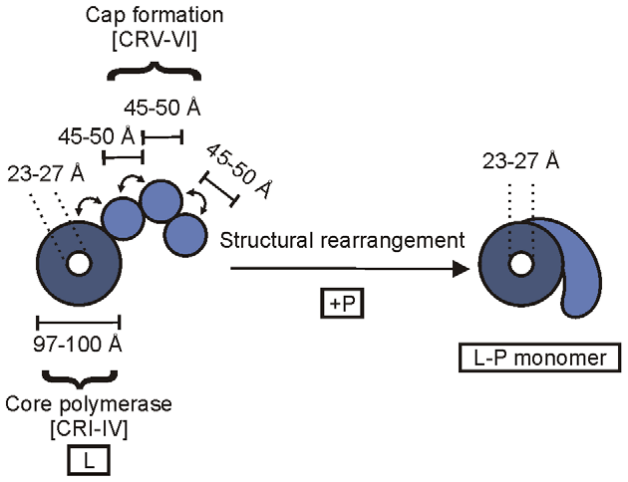
A schematic of the architecture of the VSV polymerase. A schematic depicting the arrangement of the various domains of VSV L is shown based upon electron microscopic analysis of single L protein molecules (left), along with the rearrangements that occur on complex formation with the cofactor P (right) [30]. The positions of flexibility within the L alone structure are depicted by arrows. The approximate dimensions of the protein are shown in angstroms (Å).

In combination with the recent electron microscopic structures, the present findings provide new insights into the structural and functional organization of the VSV polymerase. EM images of L alone showed the organization of L into a ring domain (90–100 Å) containing the RNA polymerase and an appendage of three globular domains (approximately 45 Å each) containing the cap-forming activities [30]. The capping enzyme maps to a globular domain that is juxtaposed to the ring and the cap methyltransferase maps to one of two more distal and flexibly connected globules. Notably, the position of the globules relative to one another appears rather flexible such that the methylase domain can be positioned adjacent to the RdRP. This arrangement of the capping and methyltransferase domains is consistent with the results of this study: the 5′ end of the mRNA originating from the RdRP domain requires a minimal length to reach the capping apparatus. The length of 100 Å is approximately the distance spanned by 31 nts, and is consistent with the distance between the center of the ring and the proximal globular domain in the appendage. The fact that the same length is sufficient to gain access to the MTase is consistent with the high degree of flexibility exhibited by the distal globular domains. However, it should be emphasized that our EM studies showed that complex formation with P induces a significant conformational rearrangement of L including the loss of the globular features of the appendage (Figure 7). It is this L-P complex that is the active form of the polymerase for RNA synthesis. The L-P complex, however, retains some features of the L protein alone, including a ring-like domain that presumably includes the RdRP activity and an altered appendage that presumably includes the capping machinery (Figure 7). The work presented here supports the idea that in the rearranged appendage, the capping active site is approximately 100Å from the RdRP active site, and the MTase domain is sufficiently flexible to not require further elongation of the mRNA.

Our findings also have implications for the path the RNA chain takes between the RdRP domain and the capping apparatus. Although, the length of the RNA would permit the transcript to traverse from the core ring domain to the PRNTase appended to the ring itself, as well as the flexible cap methylation apparatus of the same L molecule, we cannot exclude the possibility that the cap formation is catalyzed by an adjacent L molecule. Indeed, our electron microscopic examination of the L-P complex provided evidence for polymerase dimers, and genetic experiments with Sendai virus are consistent with the presence of at least a dimeric polymerase complex. It remains unknown, however, whether cap defective VSV polymerase molecules can complement RdRP defective VSV polymerase molecules. If indeed the RNA chain is capped by an adjacent polymerase molecule, such complementation should be achievable. Additionally, by incorporation of thio substituted nucleotides within the nascent RNA chain, it should now be possible to decipher the path that the RNA chain traverses in its transit from the RdRP domain to the PRNTase domain.

### Influence of capping events on nucleotide polymerization

During transcription, the capping activities of L are known from biochemical experiments to influence the polymerization activity. Specifically, failure to cap the mRNA leads to premature termination of transcription [7], [11]–[13], and inhibition of methylation of the mRNA cap can lead to hyperpolyadenylation [13]–[15]. We previously investigated the influence of sequence on premature termination by polymerases that are defective in cap addition [13]. Analogous to authentic termination, premature termination was favored at AU rich elements in the template and was significantly suppressed during copying of CG rich templates. This implies that the strength of a hybrid between the nascent strand and template can influence polymerase processivity. Although speculative, a relatively simple mechanism by which capping could influence termination is by regulating the stability of the hybrid between the nascent strand and template in the RdRP domain. Since the capping domain is juxtaposed to the ring, capping itself may induce a tightening of the grip of the polymerase stabilizing the hybrid between the template and nascent mRNA strand thus rendering the polymerase fully processive. The physical sequestration of the 5′ end of the nascent RNA strand within the PRNTase domain and then the cap methylation domain may itself also favor the RNA polymerization reaction becoming fully processive, so that the nascent RNA chain has elongated to the point at which the polymerase can only terminate in response to a highly specialized transcription stop sequence.

The length requirement for mRNA 5′ modification is similar to that previously reported for vaccinia virus [43], RNA polymerase II and the apparent length at which a reovirus transcript gains access to the capping enzyme [44]. In those other cases the capping enzyme is encoded by a separate polypeptide that appears associated with the RNA polymerase either physically in the case of the reovirus polymerase as a structural component of the virus capsid, or by recruitment to the phosphorylated C-terminal domain of RNA polymerase II [45]. For RNA polymerase II it has been shown that the nascent RNA chain is capped over a range of nucleotide positions, in this case the polymerase pauses prior to the act of mRNA cap addition, and the act of cap addition is linked to full polymerase elongation. We do not know whether a similar pausing event occurs during VSV transcription. The properties of the polymerase, however, do appear distinct immediately upon cap addition. This is illustrated by the fact that while we were able to efficiently stall the polymerase at positions +30, +31, +35, +36, +37, +40, +50 during transcription, stalling at positions +32, +33 and +34 result in a ladder of products that range up to 1–2 nucleotides longer than anticipated. Since the cap was just added at position +31, capping itself may render the polymerase somewhat resistant to stalling and result in the production of slightly longer transcripts.

A second possible explanation for the production of stalled transcripts that vary in their precise point of termination relates to the intrinsic ability of the polymerase to stutter on homopolymeric tracts. The best characterized slippage sequence for VSV is that of the gene-end AUACUUUUUUUG in which the AUAC element regulates the extent of stuttering by L on the U7 tract [46]. We considered that the template sequence CCGUUUGUCUUU present at positions 25–36 of the unperturbed 60-nt gene may itself favor some slippage that is enhanced by the insertion of three adenylates at positions 32, 33, and 34. Although the presence of G or C within a U tract blocks slippage during transit of polymerase across a gene-junction [46], the general A/U rich nature of the present sequence may be sufficient to destabilize the elongating polymerase complex in vitro. Definitive assessment of the precise point of termination of the stalled transcripts will require sequencing of these small RNA products.

### Implications of freeze-frame studies of VSV transcription

The ability to stall transcription at specific places on the template, through the use of engineered recombinant templates now provides us an ability to study more precisely the steps of transcription. This methodology should be readily adaptable to probe the length of the nascent RNA chain that is protected by the polymerase during transcription and to determine whether the RNA chain becomes accessible prior to mRNA cap addition. Moreover, combined with our ability to image the L-P complex by electron microscopy, this approach may permit us to examine the polymerase bound to the RNA template at various stages of transcription. Our work also has implications for understanding transcription in other nonsegmented negative-strand RNA viruses in that they likely require a minimal length of transcription prior to cap addition. We anticipate that those lengths will vary for each virus, and as outlined above may perhaps show gene-specific variation. Since the production of triphoshphate RNA itself may serve as an activator of specific cytosolic sensors of the innate immune system, what appears to be a specific and efficient position of mRNA cap addition may also serve to decrease the production of pppRNA transcripts in infected cells. Moreover, since the act of capping serves as a positive regulator of transcriptional elongation this minimal length represents a key check-point in the transcription cycle.

## Materials and Methods

### Generation of recombinant viruses

Plasmid pVSV1(+)60, containing an infectious cDNA clone of the VSV genome with a 60-nt long non-essential gene inserted at the leader-N gene junction, was generated as described previously [41]. The adenylates at positions 19, 20, 22, 23, 25, 28, 29 and 37-nt in the VSV genome were replaced using site-directed mutagenesis to generate an (A-) leader region (Figure 1A). In order to introduce three adenylates at positions 11, 21, 31, 32, 33, 34, 35, 36, 37, 38, 41 and 51 in the 60-nt long non-essential gene site-directed mutagenesis was performed. The presence of the mutation was confirmed by sequence analysis. Recombinant VSV was rescued from cDNA by transfection of BHK-21 cells infected with a recombinant vaccinia virus (vTF7-3) that expressed T7 RNA polymerase as described previously [47], [48]. The generated viruses were designated rVSV(A-)-10, -20, -30, -31, -32, -33, -34, -35, -36, -37, -40 and -50, respectively. Cell culture supernatants were collected at 48 to 96 h post transfection, and virus was amplified once in BHK-21 cells. Individual plaques were isolated on Vero cells, and large stocks were generated in BHK-21 cells and purified as described previously [12]. Viral titer was determined by plaque assay on Vero cells, and protein content was measured with the Bradford reagent (Sigma Chemical Co., St Louis, MO). The (A-) leader and 60-nt gene of the purified viruses were sequenced again, and these stocks were used for *in vitro* transcription reactions.

### Transcription and analysis of viral RNA

Viral RNA was synthesized *in vitro* as described previously [31], [38]. 10 µg of the purified recombinant virus was activated by incubation with detergent for 5 min at room temperature. RNA synthesis reactions were performed in the presence of nucleotide triphosphates (1 mM ATP and 0.5 mM each of CTP and GTP). The reaction mixtures were supplemented with 20 µµCi of [α-^32^P]-GTP (3,000 Ci mmol^-1^) (Perkin-Elmer, Wellesley, MA). Total RNA was extracted, purified, and used for secondary manipulations as follows. Where indicated, the RNA cap structure was removed by tobacco acid pyrophosphatase (TAP; Epicenter) as previously described [6]. For exonuclease digestion, total RNA was dephosphorylated using Antarctic phosphatase (New England Biolabs [NEB]), and a single phosphate was added using T4 polynucleotide kinase (NEB) according to the manufacturer's instructions. The resulting RNAs were subsequently treated with Terminator exonuclease (Epicenter) according to the manufacturer's instructions [12]. The products of RNA synthesis were analyzed on a 6% polyacrylamide gel and visualized by a phosphorimager (GE Healthcare; Typhoon).

### N-RNA template purification

The N-RNA template was purified from rVSV(A-)-30, -31, -32 and-33 as described previously [6]. Briefly, 4 mg purified virus was disrupted on ice for 1 h in 20 mM Tris-HCl (pH 8.0), 0.1% Triton X-100, 5% glycerol, 5 mM EDTA, 3.5 mM dithioerythritol, 20% dimethyl sulfoxide, and 1.0 M LiCl. The template was recovered by centrifugation (190,000× *g*, 3.5 h) through a step gradient of 0.25 ml each of 40, 45, and 50% glycerol in TED buffer (20 mM Tris-Cl [pH 8.0], 1 mM EDTA, 2 mM dithioerythritol) supplemented with 0.1 M NaCl. The pellet was resuspended in 0.3 ml of TED buffer plus 10% glycerol and disrupted on ice, except that the Triton X-100 and EDTA concentrations were reduced to 0.05% and 1 mM, respectively. The N RNA was isolated by banding in a 3.6-ml 20 to 40% (wt/wt) CsCl gradient (150,000× *g*, 2.5 h), recovered by side puncture and diluted fourfold with 10 mM Tris-Cl (pH 8.0), 0.1 mM EDTA. The N-RNA was recovered following centrifugation (150,000× *g*, 1.5 h) through a 0.5-ml cushion (50% glycerol, TED buffer, 0.1 M NaCl).

### Expression and purification of recombinant VSV polymerase

Recombinant L was expressed from recombinant baculoviruses in *Spodoptera frugiperda* 21 cells, and P was expressed in BL21 (DE3) as described previously [6]. At 72 h postinfection, the cells were collected, washed twice with ice-cold phosphate-buffered saline, and recovered by centrifugation. The cells were suspended in lysis buffer (50 mM NaH2PO4, 10% glycerol, 0.2% NP-40, 300 mM NaCl, 10 mM imidazole [pH 8.0]) supplemented with EDTA-free protease inhibitor cocktail (Roche) and 1 mM phenylmethylsulfonyl fluoride and disrupted by sonication. The L and P proteins were purified by Ni-nitrilotriacetic acid–agarose (Qiagen), followed by ion-exchange chromatography as described previously [6].

### Reconstitution of viral RNA synthesis *in vitro*

Reactions were carried out in the absence of rabbit reticulocyte lysate using 5 µg of N-RNA template, 4 µg of purified L, 2 µg of purified P and nucleoside triphosphates (1 mM ATP and 0.5 mM each of CTP and GTP) as described previously [6]. The reaction mixtures were supplemented with 20 µCi of [α-^32^P]GTP (3,000 Ci mmol^−1^) (Perkin-Elmer, Wellesley, MA). After 5 h of incubation at 30°C, the RNA was purified by phenol and chloroform extraction and analyzed on 6% polyacrylamide gel and visualized by a phosphorimager (GE Healthcare; Typhoon). For methylation of the transcripts, the same reaction condition was used as described above with the exception that 20 µM ^3^H-labeled S-adenosyl-L-methionine (^3^H-SAM) (76 Ci/mmol, Perkin-Elmer, Wellesley, MA) was used for labeling the RNA instead of [α-^32^P]GTP. After 5 h of incubation at 30°C, the RNA was purified by phenol and chloroform extraction and analyzed on 20% polyacrylamide gel followed by autoradiography.
